# Simple, Fast and Convenient Magnetic Bead-Based Sample Preparation for Detecting Viruses via Raman-Spectroscopy

**DOI:** 10.3390/bios13060594

**Published:** 2023-05-30

**Authors:** Susanne Pahlow, Marie Richard-Lacroix, Franziska Hornung, Nilay Köse-Vogel, Thomas G. Mayerhöfer, Julian Hniopek, Oleg Ryabchykov, Thomas Bocklitz, Karina Weber, Ralf Ehricht, Bettina Löffler, Stefanie Deinhardt-Emmer, Jürgen Popp

**Affiliations:** 1 Abbe Center of Photonics, Institute of Physical Chemistry, Friedrich Schiller University Jena, Helmholtzweg 4, 07743 Jena, Germany; susanne.pahlow@uni-jena.de (S.P.); thomas.mayerhoefer@uni-jena.de (T.G.M.); julian.hniopek@uni-jena.de (J.H.); oleg.ryabchykov@uni-jena.de (O.R.); thomas.bocklitz@uni-jena.de (T.B.); karina.weber@leibniz-ipht.de (K.W.); ralf.ehricht@leibniz-ipht.de (R.E.); 2 Center for Applied Research, InfectoGnostics Research Campus Jena, Philosophenweg 7, 07743 Jena, Germany; 3 Leibniz Centre for Photonics in Infection Research (LPI), Leibniz Institute of Photonic Technology, Albert-Einstein-Straße 9, 07745 Jena, Germany; marie.richard-lacroix@uni-jena.de; 4 Leibniz Centre for Photonics in Infection Research (LPI), Institute of Medical Microbiology, Jena University Hospital, Am Klinikum 1, 07747 Jena, Germany; franziska.hornung@med.uni-jena.de (F.H.); nilay.koese-vogel@med.uni-jena.de (N.K.-V.); bettina.loeffler@med.uni-jena.de (B.L.); 5 Physics & Computer Science, Faculty of Mathematics, Institute of Computer Science, University Bayreuth, Universitätsstraße 30, 95447 Bayreuth, Germany

**Keywords:** viruses, SARS-CoV-2, Raman spectroscopy, magnetic beads, sample preparation

## Abstract

We introduce a magnetic bead-based sample preparation scheme for enabling the Raman spectroscopic differentiation of severe acute respiratory syndrome coronavirus type 2 (SARS-CoV-2)-positive and -negative samples. The beads were functionalized with the angiotensin-converting enzyme 2 (ACE2) receptor protein, which is used as a recognition element to selectively enrich SARS-CoV-2 on the surface of the magnetic beads. The subsequent Raman measurements directly enable discriminating SARS-CoV-2-positive and -negative samples. The proposed approach is also applicable for other virus species when the specific recognition element is exchanged. A series of Raman spectra were measured on three types of samples, namely SARS-CoV-2, Influenza A H1N1 virus and a negative control. For each sample type, eight independent replicates were considered. All of the spectra are dominated by the magnetic bead substrate and no obvious differences between the sample types are apparent. In order to address the subtle differences in the spectra, we calculated different correlation coefficients, namely the Pearson coefficient and the Normalized cross correlation coefficient. By comparing the correlation with the negative control, differentiating between SARS-CoV-2 and Influenza A virus is possible. This study provides a first step towards the detection and potential classification of different viruses with the use of conventional Raman spectroscopy.

## 1. Introduction

Viruses can be defined as selfish mobile genetic elements consisting of a nucleic acid and a protective protein shell. They are, in most cases, not categorized as living organisms as they are not capable of replicating on their own and require the cell machinery of their hosts. Viruses are often able to infect more than one species as this is advantageous in terms of spreading and preserving the genetic information in the gene pool over time [1,2]. Despite their structure, which is low in complexity, and their dependence on their hosts, they are fast, highly adaptable and can successfully diversify through random and frequently occurring mutations and recombination [3]. The recent Corona Virus Disease 2019 (COVID-19) pandemic has again illustrated how fast and devastating the outcome of a zoonotic virus can be as it jumped across the species barrier [4]. Unfortunately, such crossings of the species barriers occur quite frequently and are a natural phenomenon. Modern life style changes, globalization and the destruction of natural environments promote the occurrence of such events because they lead to more intense contact between humans and different carriers of potentially infectious viruses [5]. Accordingly, the emergence of highly contagious viruses is a constant threat and scientists warn constantly that even worse scenarios are likely in the future. Continuous research aiming for the development of rapid, fast, economic, adaptable, specific, sensitive and simple diagnostic assays, together with antiviral drugs and vaccines, are key components for the preparation for, and prevention of, future outbreaks and for limiting the subsequent damage.

The current gold standard for enabling high specificity and sensitivity for diagnosing viral infections are Polymerase Chain Reaction (PCR)-based methods, including RT-PCR (reverse transcription PCR), by which the virus species or strains are identified according to characteristic nucleic acid sequences [6,7]. Despite these important advantages and their widespread application, there are some drawbacks that should not be overlooked. To date, PCR assays have remained expensive and require specialized laboratory settings with trained personnel and infrastructure. Especially in cases of a pandemic, it is crucial to consider that a global surveillance strategy, which is applicable with minimal resources, is needed. Lateral flow tests are a simple and fast detection method. Unfortunately, the sensitivity of such tests is usually considerably lower compared to PCR-based methods [8]. This highlights the need for the development of low cost and simple technologies with the potential to be applied in a decentralized way.

Spectroscopic methods appear to be a promising option as they are fast, applicable on site and give access to a wealth of information on the sample of interest. Raman spectroscopy is highly specific in terms of probing the molecular structure. It is generally considered a valuable tool for identifying substances according to their characteristic spectrum, which is sometimes referred to as their spectroscopic fingerprint. This concept can also be applied to complex biological molecules or even whole cells and bacteria [9,10]. However, Raman spectroscopy is limited by its low sensitivity, which makes virus detection quite challenging. As viruses are typically small (25–400 nm, [11]) in comparison to the excitation wavelength, large quantities are required in order to obtain pure spectra. Usually, the virus particles are embedded in a sample matrix with numerous other (Raman-active) components as they are raised in a cell culture or stem from a patient specimen, i.e., a bodily fluid. The other components (for example, proteins, nucleic acid, inorganic salts, buffer compounds) will also contribute to the collected Raman spectra and can hamper an identification based on specific bands, if no purification steps are applied. Such sample preparation can also be a challenging and costly task and is accompanied by a large amount of partly manual laboratory work. The application of advanced techniques such as ultracentrifugation [12], ultrafiltration [13] or affinity chromatography [14] can be time-consuming and require great expertise and/or expensive devices, as well as a lab with an appropriate biosafety standard. Therefore, other detection schemes are often applied in combination with Raman spectroscopy, which do not require purified virus samples. Instead of detecting the virus particles themselves, the presence of specific antibodies, nucleic acids [15] or a change of the overall composition of a certain body fluid [16,17] can be used as leverage points. The combination of those strategies with statistics-based analysis methods, such as chemometrics, then enables sample classification if the database is large enough. Quite a few approaches are based on surface-enhanced Raman spectroscopy (SERS), which employs metallic nanoparticles or nanostructures for improving the technique’s sensitivity through plasmonic enhancement [18,19,20]. As the physical phenomenon is, by definition, highly surface-sensitive, unspecific absorption must be avoided. The metallic substrates or particles can be functionalized with specific molecules (i.e., antibodies or proteins) and serve as capture elements and/or their structure can also function as a size-selective enrichment tool [21]. For example, Li et al. developed a SERS biosensor by decorating magnetic microparticles both with Au nanoparticles and the ACE2 receptor as selective capture elements for SARS-CoV-2 [22]. These particles enable the virus to be isolated from the sample matrix, and when combined with an Au nanoneedle array, it allows for a highly sensitive detection of SARS-CoV-2 from nasal swabs. Another possibility to characterize viruses is to focus on their protein structure. Sanchez et al. observed that the SERS spectra of SARS-CoV-2 are dominated by protein contributions [23]. A spectroscopic investigation of pure virus proteins might bring even deeper insight into the changes that occur on the molecular level upon the mutation of virus strains. However, some of these sophisticated approaches also come with complex issues of reproducibility and thermal stability, and are thus not yet suitable for routine use and mass production.

**Figure 1:**
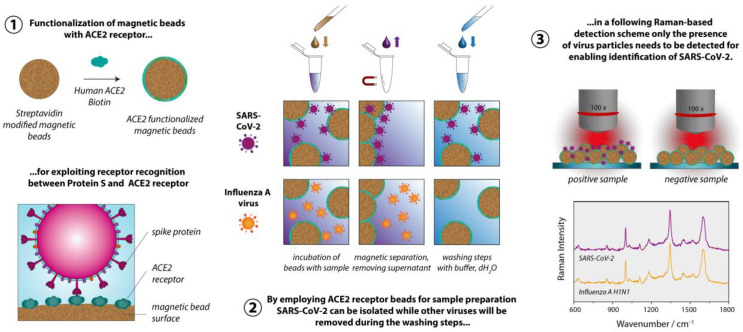
Overview of magnetic bead-based sample preparation scheme for identifying SARS-CoV-2 via Raman spectroscopy. ① First streptavidin modified beads are functionalized with biotinylated ACE2. ② Subsequently, the ACE2beads are employed to specifically capture SARS-CoV-2, while Influenza A virus or other viruses, which are not able to recognize the ACE2 receptor, are removed during the washing steps. ③ Finally, the Raman spectra of the samples are recorded.

We aimed at developing a strategy that allows viruses to be detected using conventional Raman spectroscopy, i.e., without the requirement for plasmonically active SERS substrates [18,19,20]. The summary of the proposed sample preparation scheme is displayed in Figure 1. For significantly reducing the difficulty of the task to identify a virus only based on its Raman signature, we implemented a specific capture probe in the sample preparation process, so that, ultimately, only the presence of the virus particles needs to be verified via the Raman spectrum. For the sample preparation, we established a magnetic bead-based assay, leading, simultaneously, to both highly specific virus binding and surface-to-volume optimization in comparison to flat surfaces. The magnetic beads are convenient, easily handled, imply minimal technical requirements and ensure that larger quantities of the virus are potentially present, and thus detected, in the focal plane. The functionalization of the magnetic beads with the capture probes is achieved by exploiting the biotin streptavidin interaction, which is a well-established, simple and easily reproducible procedure. Furthermore, the sample preparation method is also fully compatible with the PCR-based detection that serves as the validation methodology.

The Raman measurements are performed directly on the magnetic bead substrate after the sample preparation procedure. Accordingly, the spectra are dominated by the polymer matrix of the magnetic beads, and the spectra of the different sample types are very similar. For demonstrating the feasibility of our approach, we chose the novel coronavirus SARS-CoV-2 as the target and the Influenza A virus as the non-match control. For enabling differentiation based on the very subtle differences in the spectra, we calculated 1D correlation coefficients, such as the Pearson coefficient [24,25] and the normalized cross correlation (NCC) coefficient [25,26]. We systematically included a negative control sample in the experiments and used the respective spectra as a reference for calculating the correlation coefficients for SARS-CoV-2 and Influenza A virus. The correlation coefficients are a measure for similarity/dissimilarity, and by calculating the values relative to the negative control, we can distinguish between SARS-CoV-2 and Influenza A virus using Raman spectroscopy.

## 2. Methods

### 2.1. Preparation of ACE2 Beads

For preparing ACE2 beads, we used 50 µL (c = 10 mg/mL) of streptavidin functionalized dynabeads (M-280 Streptavidin, ThermoFisher Scientific, Waltham, MA, USA) per sample. Typically, we prepared enough beads for three samples in one batch and used 150 µL of magnetic beads accordingly. Throughout the entire protocol, 1.5 mL LoBind protein tubes by Eppendorf (Hamburg, Germany) were used. All buffers were sterilized using a syringe filter (KH54.1, Carl Roth, Karlsruhe, Germany) with a pore size of 0.22 µm. The following instructions refer to the preparation of three samples in one batch: Prior to adding the biotinylated ligand, the beads were washed twice with 500 µL 1× Phosphate Buffered Saline (1× PBS, pH 7.3, 137 mM NaCl, 2.7 mM KCl, 4.3 Na_2_HPO_4_, 1.4 mM KH_2_PO_4_). The beads were resuspended in 150 µL 1× PBS, and 13.5 µg biotinylated ACE2 (=4.5 µg per sample) was added. Biotinylated Human ACE2 (AC2-H82E6) was purchased from Acro Biosystems, Newark, DE, USA. The total volume was then adjusted to 200 µL by adding more 1× PBS. The sample was incubated for 30 min at room temperature under end-over rotation (12 rpm) to ensure proper mixing. Subsequently, the beads were washed twice with 500 µL 1× PBS and twice with 500 µL 0.05 M PIPES (1,4-Piperazinediethanesulfonic acid) + 0.1 M NaCl, pH 6.5. The beads were finally resuspended in 155 µL of PIPES buffer and 50 µL per sample were pipetted into new vials.

### 2.2. Virus Isolation

For virus isolation, 200 µL of virus culture, or in cases of the negative control, just the cell medium, was added per vial. The samples were incubated for 60 min at room temperature using end-over rotation (12–15 rpm). The beads were then washed twice using 500 µL of PIPES buffer and finally resuspended in 55 µL of the same buffer. The sample was then split into two equal parts of 25 µL each. One vial was used for the PCR detection scheme, the other one was inactivated by adding 4% of paraformaldehyde (PFA) to the bead solution for 30 min at 37 °C, and then further processed for the Raman detection scheme, as described in the next paragraph.

### 2.3. Raman Measurements

Prior to the Raman measurements, the magnetic beads were quickly washed twice with 100 µL of dH_2_O and then resuspended in 50 µL dH_2_O. Three 1 µL droplets per sample were applied to a Raman compatible substrate. We used silicon chips with an aluminum layer. Their detailed fabrication process can be found in [27]. The dried droplets were investigated using a KAISER Raman microscope with a 100× LWD objective with NA = 0.75. The excitation wavelength was 808 nm with a power at the sample adjusted to 2.5 mW and an acquisition time per spectrum of 60 s.

### 2.4. Data Processing 

RAMANMETRIX (version 0.3.5) [28] was used for data preprocessing. The Raman spectra were despiked and wavenumber calibrated using 4-Acetamidophenol as reference substance. Interpolation onto the linear wavenumber axis with a step size of 1 cm^−1^ was conducted in the range between 200–3105 cm^−1^. A second order SNIP baseline correction with smoothing and 40 iterations, as well as vector normalization, was conducted. The correlation coefficients were calculated with Wolfram Mathematica (version 12.3) using the wavenumber range 201–1749 cm^−1^. The Kolmogorov-Smirnov-Test was performed using GNU R 4.0.3, using the ks.test function provided by the stats packages with the null hypothesis CDF_SARS-CoV-2_ > CDF_Influenza_ (function parameter: alternative = “less”) [29].

### 2.5. Cell Culture and Viral Propagation

The viral propagation of SARS-CoV-2 was performed with the human lung cancer cell line Vero-E6, a gift from the Charité Berlin. Cells were cultured in EMEM (Eagle’s Minimum Essential Medium, ATCC, Wesel, Germany) with HEPES and 5 mM L-glutamine at 5% CO_2_ in 37 °C. For the infection, we used a viral isolate from a respiratory specimen of a COVID-19 patient (SARS-CoV-2/hu/Germany/Jenavi005587/2020, ethics approval of the Jena University Hospital, no. 2018–1263). The determined sequence is available in the NCBI GenBank under the accession number MW633323, and further information on the strain can be found in [30]. The isolation and propagation of Influenza A virus (IAV) was performed on Madin-Darby canine kidney cells (MDCK) cultured in EMEM and 10% FCS at 5% CO_2_ in 37 °C. The patient isolate IAV/H1N1/vi013320 was achieved by using a standard protocol containing plaque assay purification, as described previously [30].

### 2.6. Virus Lysis and qRT-PCR

After performing the virus isolation with the magnetic beads, RLT lysis buffer (Qiagen, Hilden, Germany) was added to the vial containing the sample for the qPCR analysis. The viral (v)RNA of SARS-CoV-2 lysates was extracted by using the QIAcube RNeasy Viral Mini Kit (Qiagen, Hilden, Germany) according to the manufacture’s guidelines. The detection of the SARS-CoV-2-specific RNA was performed using the RIDAgene Kit (r-biopharm, Darmstadt, Germany) with the Rotor-Gene Q (Qiagen, Hilden, Germany). This kit detects the E-gene of SARS-CoV-2 and allows the quantification of the number of copies by a given positive control. For the extraction of the vRNA of IAV, we used the EZ1 Virus Mini Kit v2.0 (Qiagen, Hilden, Germany). The detection of the Influenza A specific M2 gene via qRT-PCR was carried out with the LightMix Modular Influenza A (InfA M2) Kit from TIB Molbiol (Berlin, Germany) with the LightCycler 480 (Roche, Mannheim, Germany).

## 3. Results and Discussion

As depicted in Figure 1, our proposed Raman-based detection strategy to identify virus particles rests on a highly specific sample preparation scheme. To selectively enrich the beads’ surface with virus particles of the virus type to be detected, a specific recognition element is required. Instead of employing an antibody, we decided to exploit the ACE2 receptor that SARS-CoV-2 uses for attaching itself to the host’s cells [31,32]. As is the case in all coronaviruses, SARS-CoV-2 uses its spike glycoprotein (S protein) for binding to the cellular receptors [33]. The particularly specific binding between the viral S protein and the host receptor is also responsible for the efficient transmission of SARS-CoV-2 among humans [34]. However, this is only the first step of the viral entry into the cells. The trimeric S protein has a clove-like structure, with a S1 (head) and a S2 (stem) subunit. While the S1 unit contains the receptor binding motif, the S2 unit is responsible for the fusion with the host cell membrane, after which the nucleic acid is inserted into the cells. For this process, proteases such as furin or transmembrane serine protease 2 (TMPRSS2) are required for cleaving the S protein at distinct sites [35]. Employing the receptor instead of an antibody as the recognition element is advantageous from our perspective as viruses are constantly subject to mutation, and therefore alter their protein structure. Accordingly, certain mutations and/or recombination will cause an antibody-based assay to fail because the target element is poorly or no longer recognized. By using the very same element as the capture probe that the virus needs to infect the cells, such an issue will most likely not occur, even when the virus mutates and recombines [36].

As mentioned before, we aspire to detect the virus particles with conventional Raman spectroscopy. Due to the small size of the virus and potentially low concentrations, we needed to enrich them before conducting the actual measurements. To that end, we employed magnetic beads-micrometer-sized polymer particles with magnetic nanoparticles embedded in them. They are commercially available with various surface modifications, enabling different strategies for functionalization. We chose streptavidin-modified magnetic beads as their further decoration with capture elements is very easy and well reproducible. The immobilization of the capture probes on such surfaces exploits the very strong interaction between the protein avidin or streptavidin (an avidin protein produced by bacteria of the genus *Streptomyces*) and the vitamin biotin. With a dissociation constant of K = 10^−15^ M, this biological interaction is the strongest known non-covalent interaction [37]. Further advantages are the relatively high robustness in terms of the pH, temperature and solvents [38]. Many biomolecules can be purchased with biotin modification or, alternatively, coupling kits are available, which also allow non-experts to perform a biotinylation. In addition, applying the proposed sample preparation concept comes with very few obstacles. In our study, we used a prefabricated biotinylated ACE2 receptor. 

**Figure 2:**
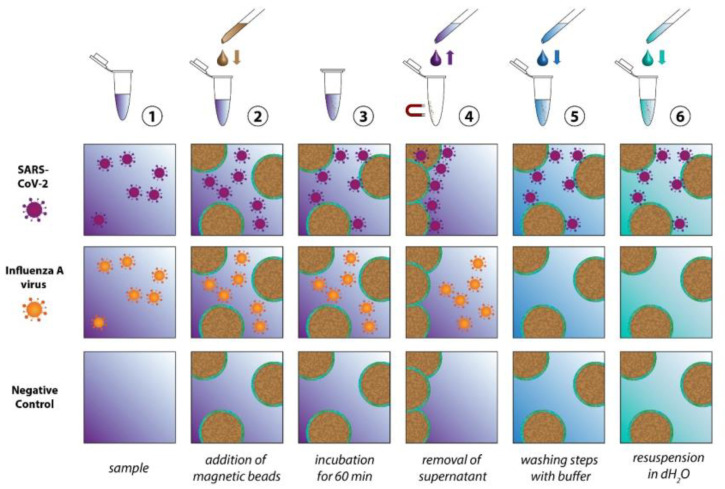
Detailed display of the magnetic bead-based sample preparation scheme. To the sample ① the ACE2 functionalized beads ② are added. After a 60 min incubation period ③, the supernatant is removed ④ and the sample is washed with buffer and dH_2_O ⑤ and finally resuspended in dH_2_O ⑥.

The scheme in Figure 2 depicts the individual steps of the sample preparation procedure. In the first step, the ACE2-functionalized particles are added to the sample, which might contain SARS-CoV-2, another virus (for example Influenza A virus), other microorganisms or no pathogens at all. During the incubation time, the SARS-CoV-2 particles will attach themselves to the bead surface due to the spike protein receptor, with a reported binding constant ranging between 0.11 nM [39] and 14.7 nM [40]. In order to achieve the selective enrichment of SARS-CoV-2, washing steps are implemented, where the solid phase (the magnetic particles) is separated from the liquid phase (the supernatant). During these washing steps, all viruses that are not immobilized on the bead surface will be removed. The same happens to all other components of the samples that are in the liquid phase. Through repetition of the washing steps, not only is the selection of the virus species of interest achieved, but also the complexity of the sample is significantly reduced. In the final step, all three sample types essentially consist of the washing buffer, the magnetic beads and, in the case of the SARS-CoV-2 sample, the virus particles. To enable the Raman detection on a dried sample, a further washing step with dH_2_O is implemented to remove the buffer salts. For this study, inactivated SARS-CoV-2 viruses were used. Further information is provided in Section 1 (and associated Figure S1) of the Supporting Information. 

**Figure 3:**
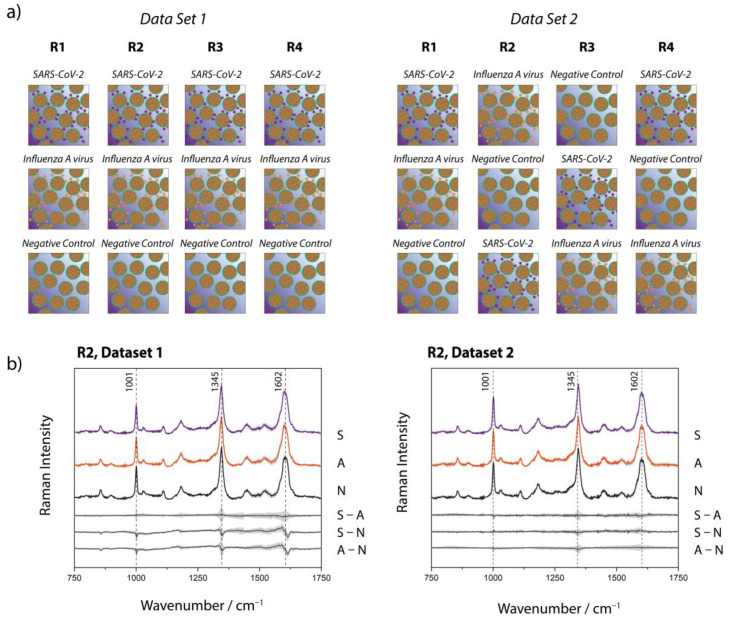
(**a**) Structure of the data sets used for the Raman spectroscopic investigation. For each data set four independent replicates (R1–R4) were considered. (**b**) Raman mean spectra of SARS-CoV-2 (S), Influenza A virus (A) and the negative control (N) as well as the difference spectra. The spectra were shifted vertically for clarity.

Figure 3a shows the Raman-based identification scheme, for which two data sets have been investigated, each consisting of four replicates per sample type (SARS-CoV-2, Influenza A virus, negative control). The replicates were processed and measured on independent days. It is noteworthy that the two data sets have been investigated several months apart to ensure the reproducibility of the study under different environmental conditions (ex. season change creating variating humidity index, slight lab temperature variation, new batches of magnetic beads, etc.). Two significant differences have been introduced voluntarily: (1) For data set 1, the ACE2 functionalized beads were prepared in one batch, while for data set 2, a fresh batch of ACE2 beads has been prepared for each replicate; (2) As shown in Figure 3a, the order in which the samples were measured within an experimental day has been varied for data set 2, with the objective of ruling out the potential impact of any minor optic changes that can occur in the course of an experimental day.

During the Raman measurements, we also observed that the SARS-CoV-2 samples often looked microscopically different from the other sample types. As shown in the microscopic images of Figure S4, the magnetic beads form large agglomerates for the SARS-CoV-2 samples, while for the other sample types, this effect is much less pronounced, if it occurs at all. This formation of large clusters can be ascribed to the multiple binding sites that are present both on the spherical virus particles and on the ACE2 decorated magnetic beads. In principle, this effect alone has great potential for detecting the presence of SARS-CoV-2 using a simple assay. However, we found that the extent of this cluster formation with the current workflow can differ significantly and is therefore not a reliable criterion for identifying SARS-CoV-2 by itself. The Raman measurements were conducted with an incident irradiation of 808 nm, which remains significantly under the typical size of a single bead (2.8 μm in diameter). The agglomeration of the beads (domain typically composed of a few tens to several hundreds of beads) does not appear to create additional scattering that could modify the background of the spectra (vide infra). However, to ensure the absence of interferences in the data due to this phenomenon, a minimum of 49 spectra were acquired at different locations on the sample and where the morphology of the bead arrangement differed.

Figure 3b displays, as a representative example, the mean Raman spectra of one subset of each data set (all mean and standard deviation spectra are shown in Figure S2 of the Supporting Information), where each set of data consists of 49 to 138 single spectra per sample type, measured with an acquisition time of 60 s. The spectra of the different sample types (SARS-CoV-2, Influenza A virus, negative control) are virtually undistinguishable. The subtracted spectra in Figure 3b and Figure S2) expose even more clearly the absence of significant differences in the average spectra of the investigated sample types. The variations observed at ~ 1001, 1345 and 1602 cm^−1^ are all approximately within the standard deviation, making any deeper analysis of the spectral differences based on those data highly arbitrary. It needs to be pointed out that, even when these spectral changes were significant, one still needs to interpret these results with great caution as many bands occur for almost all proteins and are not necessarily characteristic for the SARS-CoV-2 proteins. The Raman signal for all sample types is mainly dominated by the magnetic bead substrate (see Figure S3 of the supporting information, where a reference spectrum acquired from the non-functionalized streptavidin magnetic beads can be found, along with a spectrum of the ACE2). The beads themselves consist of a polystyrene-divinylbenzene (PS-DVB) matrix [41] with magnetite [42] and maghemite as magnetic material and are functionalized with streptavidin and the ACE2, which are both proteins. We originally hypothesized that differentiating between the SARS-CoV-2 and Influenza samples would be enabled by an enhanced protein signature in the Raman spectra of the beads incubated with the SARS-CoV-2 particles. However, the qRT-PCR results (Table S1) indicate that, to some minor extent, Influenza A viruses also unspecifically adsorb onto the surface of the ACE2 functionalized beads. It is noteworthy that the differences between the SARS-CoV-2 and Influenza A virus with the negative control are not more pronounced, illustrating that the protein contribution to the signal must be minor, if at all detectable. This is likely due to the small amount of virus attached to the beads in combination with the low sensitivity of the Raman signal. Increasing the acquisition time or the power at the sample for signal optimization (or noise reduction) unfortunately leads to observable damage, most likely due to the absorption of the 808 nm wavelength by the beads (data not shown). Our attempts to use 532 nm, and even 785 nm, incident wavelengths (to have access to a higher signal-to-noise ratio) have failed due to the strong fluorescence background arising from the magnetic beads. An alternative way for detecting SARS-CoV-2 using our proposed sample preparation scheme is to detach the isolated virus particles from the magnetic beads before performing the Raman measurements. Thereby, the influence of the magnetic bead material and the protein modification (streptavidin and ACE2 receptor) can be minimized, and the spectra will be dominated by the contributions of the virus particles themselves. In contrast to our proposed method, this could enable identifying the virus particles according to their characteristic Raman signature. However, this approach requires additional working steps, and it needs to be ensured that enough viruses are retrieved for a Raman spectrum to be recorded. In cases of a highly purified sample, detailed information on the protein structure can be obtained and allows for discrimination between SARS-CoV-2 strains, as was demonstrated by Pezzotti et al. [43].

**Figure 4:**
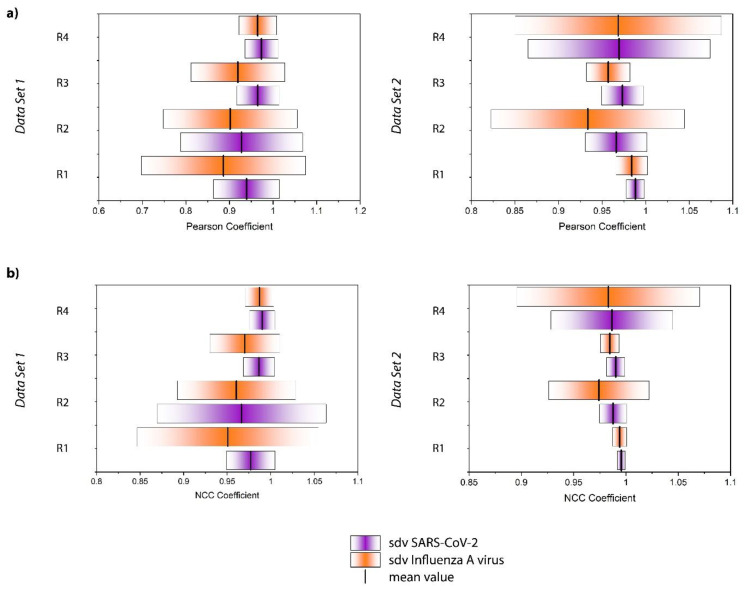
Display of the average value and standard deviation (sdv) of the (**a**) Pearson coefficient and (**b**) normalized cross correlation coefficient for each replicate (R) and data set.

Correlation coefficients enable quantifying similarities/dissimilarities between data sets. The Pearson coefficient, for example, is a measure for linear correlation. It can attain values between −1 and +1. A correlation coefficient of exactly +1 or −1 represents a perfectly linear (or inverse linear) relationship between the compared groups, while 0 indicates the absence of any linear dependency [44,45]. We calculated the Pearson coefficient, according to Equation E1 (Supporting Information), between each individual spectrum of the series of SARS-CoV-2 and Influenza A virus and the average spectrum of the series of spectra of the negative control. The outcome of this calculation for every replicate and data set is displayed in Figure S5. As expected, the values for both the Influenza A virus and SARS-CoV-2 samples are very close to 1, expressing high spectral similarity. However, a trend appears when evaluating the average of the Influenza A virus and SARS-CoV-2 samples for each replicate and data set, as displayed in Figure 4a. Although there is significant replicate-to-replicate variability, when taking every replicate individually, the SARS-CoV-2 samples are always associated with a mean value closer to +1 and tend to have a smaller standard deviation.

Moreover, we calculated the normalized cross correlation coefficient according to Equation E2 (Supporting Information). This correlation coefficient is often used in image analysis, for example, to enable pattern recognition. In contrast to the Pearson coefficient, also referred to as the zero-mean normalized cross correlation coefficient, no zero-mean centering (subtracting the average intensity) is carried out. Therefore, it has been found to be more sensitive in pattern recognition applications [25]. The results for the single spectra are displayed in Figure S6, and the average values and standard deviations are summarized in Figure 4b. The results are similar to the Pearson coefficient. All of the values are close to +1 and the SARS-CoV-2 samples result in mean values systematically larger than those of the Influenza A virus. Overall, Figure 4 reveals that the NCC coefficient seems to be slightly more sensitive than the Pearson coefficient for detecting the subtle differences in the spectra, in agreement with the findings of Martin and Crowley, perhaps because the NCC coefficient is more stable with regard to noise effects [25]. Again, the standard deviation of the SARS-CoV-2 samples tends to be smaller than that of the Influenza A virus samples. The larger standard deviation associated with the Influenza A virus sample is likely attributed to the above-mentioned unspecific binding observed through the PCR analysis. Indeed, one expects the dispersion of these particles on the surface to be much less homogeneous than that of the well-defined immobilization of the SARS-CoV-2 viruses. Accordingly, by analyzing different regions of the sample, we have probably been investigating areas that contain larger or smaller quantities of those residues, resulting in a standard deviation that is markedly higher. Furthermore, it is noteworthy that the correlation coefficients for the SARS-CoV-2 samples are always closer to +1, indicating a greater similarity to the negative control. This seems counterintuitive, as one expects the Influenza A virus samples to resemble the negative control more closely because, in contrast to the SARS-CoV-2 samples, these virus particles do not bind to the ACE2 receptor on the magnetic beads. However, this result should not be overinterpreted as the spectra are virtually identical and no distinct features can be assigned to a specific sample type. Rather than interpreting the correlation coefficients in the classical way, we conclude that the differentiation is much more likely to be due to a different signal to noise ratio and/or may be related to the less homogenous microstructure of the sample.

It is important to consider that the distribution of the correlation coefficient values for each type of sample is quite large. Nevertheless, the relative values associated with the SARS-CoV-2 and Influenza A virus samples are consistent from replicate to replicate. To further verify the validity of our hypothesis that the series are indeed statistically distinctive, a Kolmogorov-Smirnov test (KS-Test) was carried out (Table 1). The KS-Test is a non-parametric statistical test to compute the likelihood of two samples being drawn from the same probability distribution [46]. The test statistic is based on comparing the cumulative distribution functions (CDFs) of the two samples and computing the maximum distance between the two [47]. If this distance is large, the likelihood of the two samples being drawn from the same population is low, and vice versa. As this test statistic is sensitive to differences in both the location and shape of the distribution functions, it is a powerful statistical measure to quantify the distance between samples. Furthermore, as a non-parametric test, the KS-test does not assume a specific distribution function, i.e., normality is not required as, for example, in the case of Student’s t-test. Using the one-sided variant of the KS-test with null hypothesis CDF_SARS-CoV-2_ > CDF_Influenza A virus_ shows that, for the majority of the samples, the correlation values for the SARS-CoV-2 samples are larger than those for Influenza on a statistically significant level (α = 0.05), thereby strongly supporting the hypothesis that higher values for the correlation coefficients coincide with the detection of SARS-CoV-2. In general, NCC and Pearson correlations are performed similarly, and the significance of the result at α = 0.05 only changed for replicate 1 of the first data set. In replicate 2 of the first dataset, as well as in replicates 1 and 4 of the second dataset, the KS-test *p*-values for NCC and Pearson correlation are even numerically equal.

## 4. Conclusions

We have demonstrated how a simple magnetic bead-based sample preparation scheme can enable the differentiation of SARS-CoV-2 and Influenza A virus using conventional Raman spectroscopy. In contrast to several already existing approaches, our method does not need a plasmonically active SERS substrate, thereby making it easier to apply and less prone to reproducibility issues. By exploiting the specific interaction of the ACE2 receptor and the spike protein of SARS-CoV-2, we were able to selectively enrich the virus and subsequently acquire the Raman spectra of the bead-bound virus. As a non-match control, we included Influenza A virus H1N1 in our study. As the spectral differences between the SARS-CoV-2-positive and -negative samples were subtle, 1D correlation analysis was used for achieving a successful differentiation. We calculated both the Pearson and NCC coefficients for quantifying the differences between samples and found that both coefficients perform very similarly, with the NCC coefficient being slightly more sensitive. The presented approach represents a first step towards virus identification using conventional Raman spectroscopy.

## Figures and Tables

**Table 1:** Average values and standard deviations and results of the Kolmogorov-Smirnov test for SARS-CoV-2 and Influenza A virus samples, for all replicates and for both data sets (a) based on the normalized cross correlation coefficient and (b) based on the Pearson coefficient.

**(a) NCC Coefficient**				
	**SARS-CoV-2** **Mean ± Sd**	**Influenza A Virus** **Mean ± Sd**	**Mean Value**	***p*-Value** **Bold: Significant at** ***α = 0.05***
Data Set 1				
Replicate 1	0.977 ± 0.028	0.951 ± 0.104	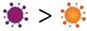	0.0261
Replicate 2	0.966 ± 0.097	0.960 ± 0.068	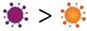	0.0889
Replicate 3	0.986 ± 0.018	0.970 ± 0.040	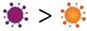	0.0340
Replicate 4	0.990 ± 0.015	0.987 ± 0.016	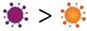	0.0154
Data Set 2				
Replicate 1	0.996 ± 0.004	0.994 ± 0.007	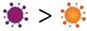	0.0258
Replicate 2	0.988 ± 0.013	0.974 ± 0.048	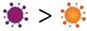	2.93 × 10^−8^
Replicate 3	0.990 ± 0.009	0.985 ± 0.009	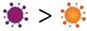	3.08 × 10^−7^
Replicate 4	0.987 ± 0.058	0.983 ± 0.088	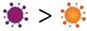	0.944
(b) Pearson coefficient				
Data Set 1				
Replicate 1	0.939 ± 0.076	0.886 ± 0.188	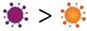	0.0682
Replicate 2	0.928 ± 0.140	0.902 ± 0.154	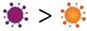	0.0889
Replicate 3	0.965 ± 0.049	0.919 ± 0.108	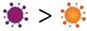	0.0198
Replicate 4	0.973 ± 0.038	0.965 ± 0.043	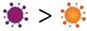	0.0154
Data Set 2				
Replicate 1	0.988 ± 0.010	0.984 ± 0.018	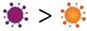	0.0258
Replicate 2	0.966 ± 0.035	0.934 ± 0.111	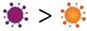	4.60 × 10^−5^
Replicate 3	0.973 ± 0.038	0.957 ± 0.025	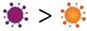	1.22 × 10^−7^
Replicate 4	0.970 ± 0.105	0.969 ± 0.118	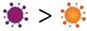	0.944
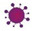 SARS-CoV-2 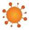 Influenza A virus				

## Data Availability

Data are available upon request.

## Supplementary Materials

The following supporting information can be downloaded at: https://www.mdpi.com/article/10.3390/bios13060594/s1, Figure S1: SARS-CoV-2 viral load determined via qRT-PCR after magnetic bead-based isolation; Figure S2: Raman mean spectra of SARS-CoV-2 (S), Influenza A virus (A) and the negative control (N); Figure S3: Raman spectra of dried ACE2 solution, ACE2 functionalized beads and plain streptavidin beads; Table S1: qRT-PCR Results; Figure S4: Microscopic images of the different sample types (SARS-CoV-2, Influenza A virus, negative control); Figure S5: Distribution of the Pearson coefficients for all SARS-CoV-2 and Influenza A virus samples, for all replicates and for both data sets; Figure S6: Distribution of the normalized cross correlation (NCC) coefficients for all SARS-CoV-2 and Influenza A virus samples, for all replicates and for both data sets.
