# Supplementary material for: Simple, Fast and Convenient Magnetic Bead-Based Sample Preparation for Detecting Viruses via Raman-Spectroscopy
Source: Biosensors (Basel). 2023 May 30;13(6):594. doi: 10.3390/bios13060594 (PMC10296004; doi:10.3390/bios13060594)
Supplement: Supplementary file 1 [file biosensors-13-00594-s001.zip › biosensors-2383996-supplementary.pdf]

Supporting Information

Section 1: Effects of the Inactivation Procedure

Because of its high contagiousness special precautions are necessary when working with SARS-CoV-2 samples. To minimize the risk for the lab personnel, we investigated, if an inactivation of the virus sample is compatible with our approach. To that end we prepared samples with active and inactivated SARS-CoV-2 and evaluated via PCR, if the recognition between the spike protein and the ACE2 receptor still works efficiently. Furthermore, we implemented negative controls to additionally verify, that the isolation of SARS-CoV-2 is actually achieved due the receptor recognition and not some unspecific adsorption. Overall, we investigated four different sample types: ACE2 modified magnetic beads 2) with virus sample and 2) without virus sample (as negative control) and plain streptavidin beads 3) with virus sample and 4) without virus sample (as negative control). For each of these conditions we either inactivated the virus particles A) after or B) before the isolation procedure. The isolation yield of the sample containing virus particles (A1, A3 and B1, B3) was determined using quantitative real time polymerase chain reaction (qRT-PCR). The results are depicted in **Figure S1**. As expected, the highest yield was achieved for sample A1. In comparison to sample A3 with the plain streptavidin beads, the ACE2 functionalized beads of sample A1 bind the viruses more efficient and achieve a yield more than one order of magnitude larger. This result, however, also shows that significant unspecific binding of SARS-CoV-2 to streptavidin beads also occurs with protocol applied. The lower yield of sample B1, where the viruses were inactivated before incubation with the ACE2 beads, suggests that the inactivation procedure with formaldehyde alters the structure of the spike protein and recognition of the ACE2 receptor is consequently hampered. Accordingly, for all following samples we performed the inactivation after completing the isolation procedure.

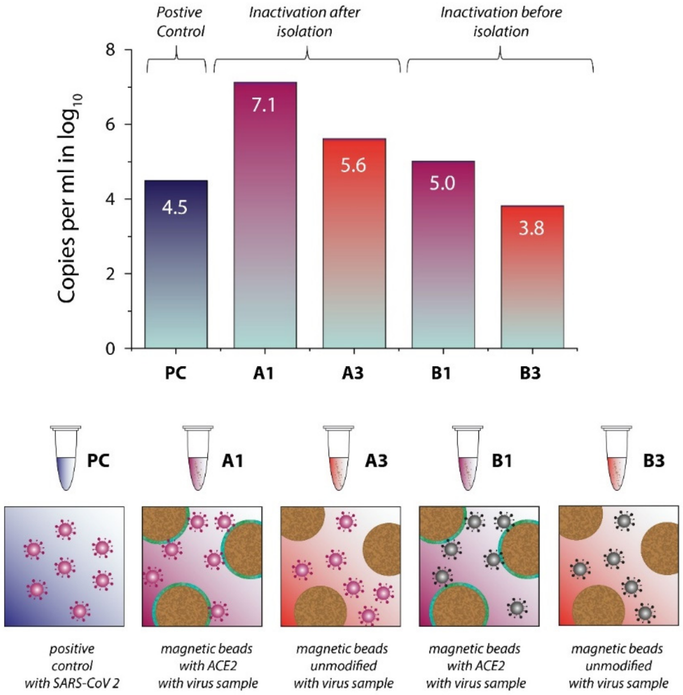

Figure S1. SARS-CoV-2 viral load determined via qRT-PCR after magnetic bead-based isolation. Samples A1 and A3 have been inactivated after the isolation procedure, while the viruses for samples B1 and B3 have been

inactivated before performing the isolation protocol. For the samples A1 and B1 the magnetic beads were functionalized with ACE2. For the samples A3 and B3 plain streptavidin beads were used.

Section 2: Raman Analysis

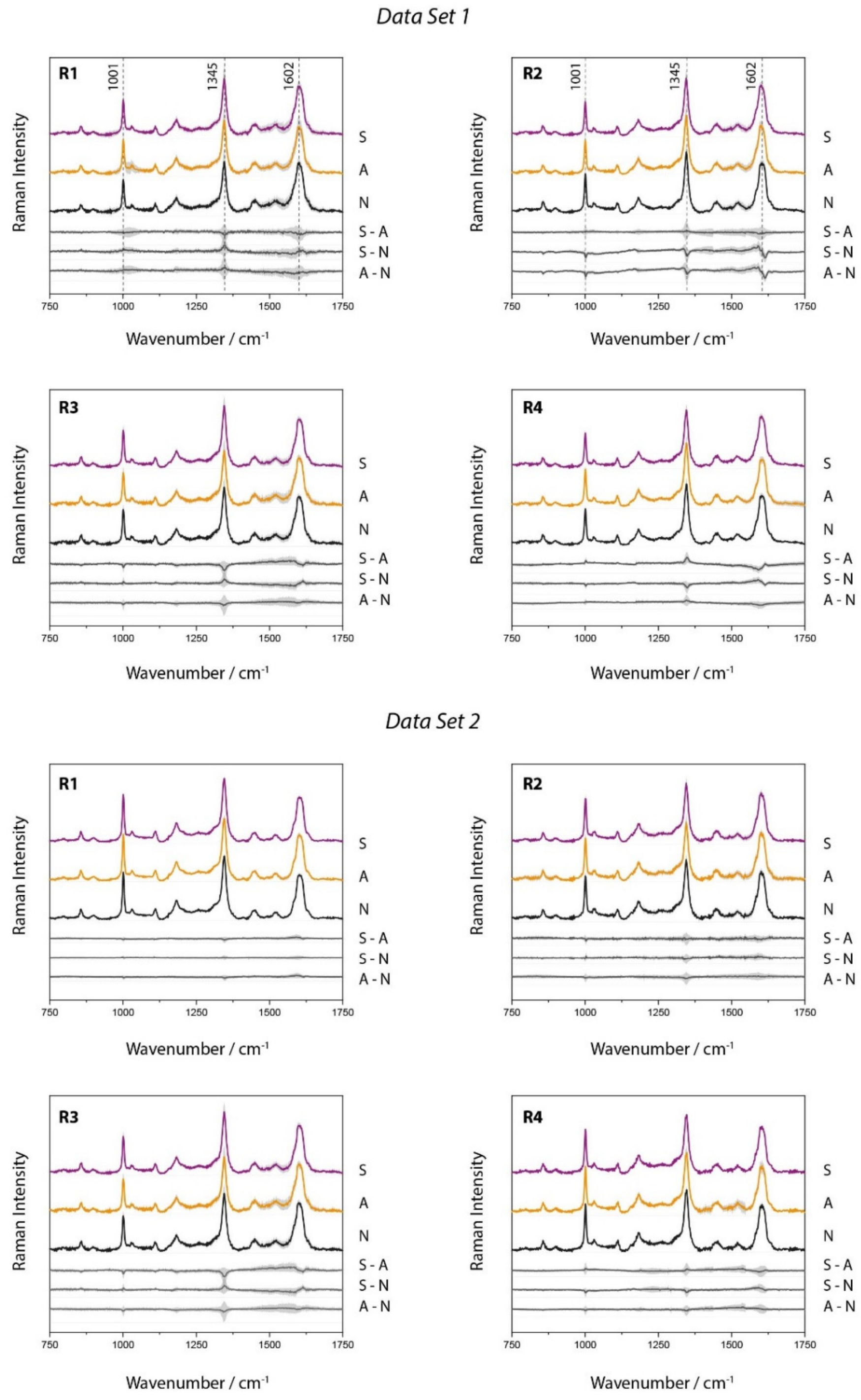

Figure S2. Raman mean spectra of SARS-CoV-2 (S), Influenza A virus (A) and the negative control (N) as well as the difference spectra for each replicate (R) for data sets 1 and 2. The spectra were shifted vertically for clarity. The standard deviation is displayed in light grey.

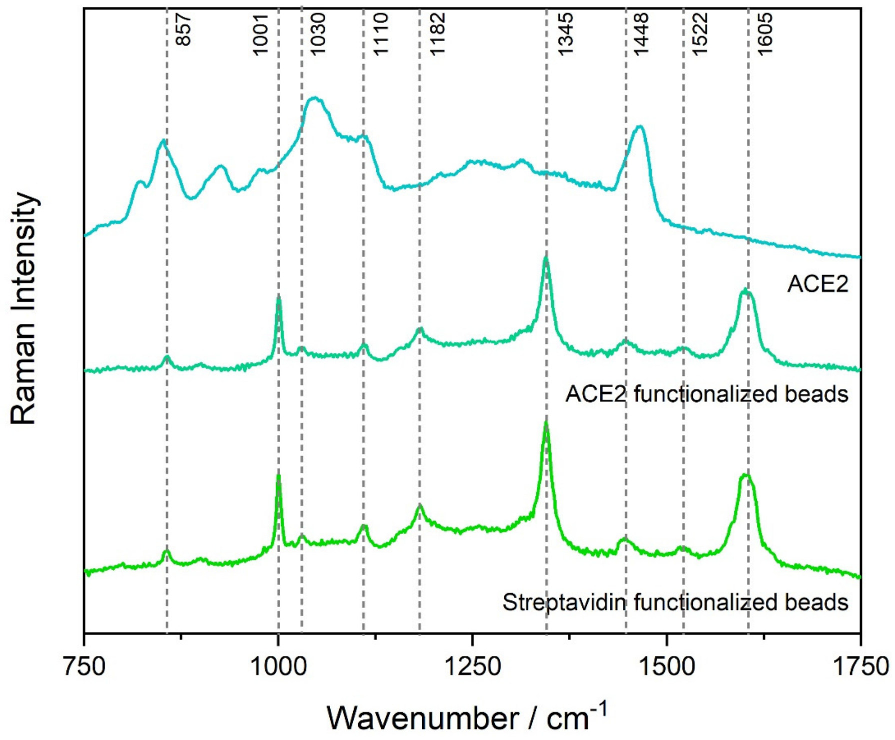

Figure S3. Raman spectra of dried ACE2 solution, ACE2 functionalized beads and plain streptavidin beads. The spectra were shifted vertically for clarity.

Section 3: PCR Results

Table S1. qRT-PCR Results.

	**Cycle threshold C_t**<br>*RIDAgene Kit for SARS-CoV-2 (r-biopharm)*	**Cycle threshold C_t**<br>*InfA M2 Kit for Influenza A (TIB Molbiol)*
**Data Set 1**		
SARS-CoV-2 (R1)	**11,115**	bld
Influenza A virus (R1)	bld	**18,7**
negative control (R1)	bld	bld
SARS-CoV-2 (R2)	**10,89**	bld
Influenza A virus (R2)	bld	**21,1**
negative control (R2)	bld	bld
SARS-CoV-2 (R3)	**11,28**	bld
Influenza A virus (R3)	bld	**20,1**
negative control (R3)	bld	bld
SARS-CoV-2 (R4)	**10,17**	nd
Influenza A virus (R4)	bld	nd
negative control (R4)	bld	nd
**Data Set 2**		
SARS-CoV-2 (R1)	**26,98**	nd
Influenza A virus (R1)	bld	nd
negative control (R1)	bld	nd
SARS-CoV-2 (R2)	**25,06**	nd
Influenza A virus (R2)	bld	nd
negative control (R2)	bld	nd
SARS-CoV-2 (R3)	**30,14**	nd
Influenza A virus (R3)	bld	nd
negative control (R3)	bld	nd
SARS-CoV-2 (R4)	**24,27**	nd
Influenza A virus (R4)	bld	nd
negative control (R4)	bld	nd

bld – below detection limit, nd – not determined

Section 4. Microscopic Images

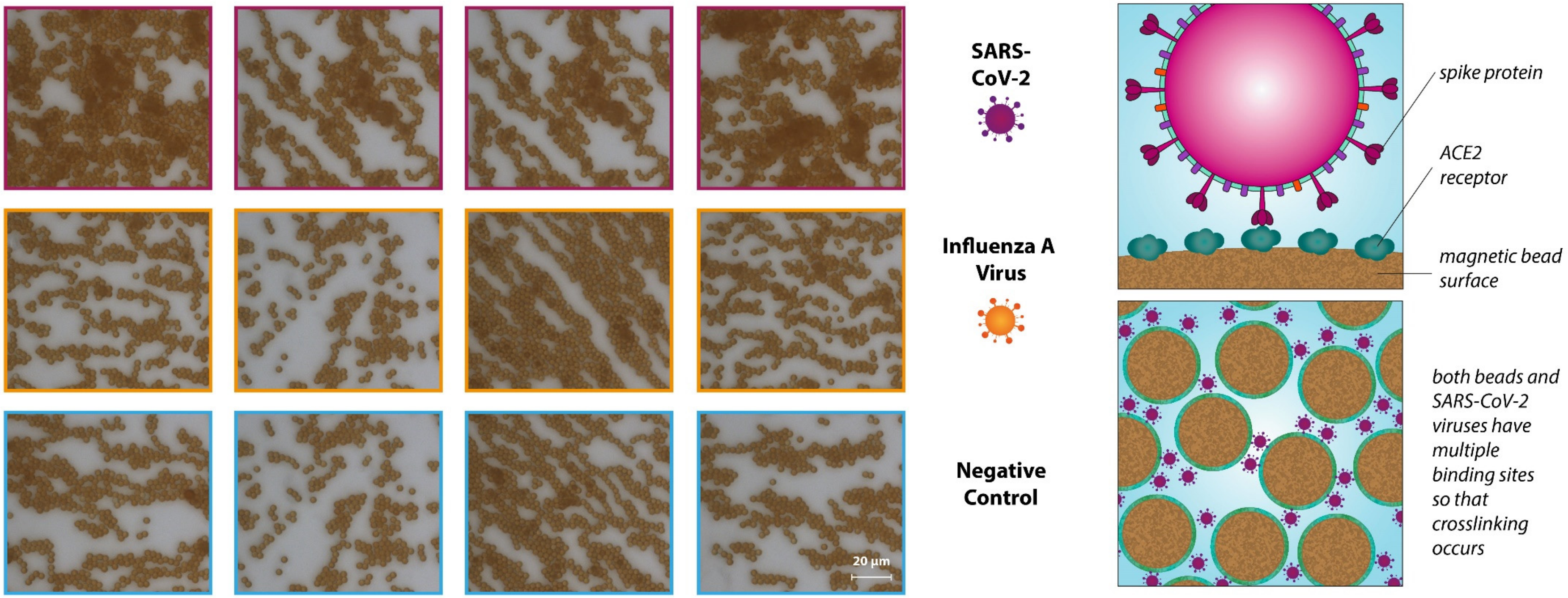

Figure S4. Microscopic images of the different sample types (SARS-CoV-2, Influenza A virus, negative control). The beads have a diameter of 2.8 µm. For the SARS-CoV-2 samples partially an agglomeration effect can be observed, which is most likely due to the multiple binding sites available on the Virus and the magnetic bead surface as schematically displayed on the right side of this figure.

Section 5. Calculation of Correlation Coefficients

Pearson Coefficient

$$r_{\text{Pearson},\,j} \;=\; \frac{1}{n-1}\sum_{i=1}^{n}\left[\left(\frac{y_i(s_j)-\overline{y}(s_j)}{\sigma(s_j)}\right)\left(\frac{y_i(s_{NK,\,average})-\overline{y}(\overline{S_{NK}})}{\sigma(\overline{S_{NK}})}\right)\right] \qquad \text{(E1)}$$

$$\sigma(s_j) \;=\; \sqrt{\frac{1}{n-1}\sum_{i=1}^{n}(y_i(s_j)-\overline{y}(s_j))^2}$$

$y_i(s_j)$: Intensity of the jth spectrum (s_j) at wavenumber i

$\overline{y}$ (s_j): Average intensity of the jth spectrum (s_j) over the whole wavenumber range

s_j: single spectrum from one sample (SARS-CoV-2 or Influenza) of a specific batch (Data Set 1 OR Data Set 2) from a specific replicate (day R1 – R4)

$$\sigma(\overline{S_{NK}}) \;=\; \sqrt{\frac{1}{n-1}\sum_{i=1}^{n}(y_i\left(\overline{S_{NK}}\right)-y_{average}\left(\overline{S_{NK}}\right))^2}$$

$y_i(\overline{S_{NK}})$: Intensity of the average negative control spectrum ($s_{NK,\,average}$) at wavenumber i

$\overline{y}$ ($s_{NK,\,average}$): Average intensity of the average negative control spectrum spectrum $\overline{S_{NK}}$ over the whole wavenumber range

$\overline{S_{NK}}$: average negative control spectrum, we always use the negative control from the same batch and day, that the spectrum s_j was taken from

Normalized Cross Correlation Coefficient

$$r_{\text{NCC},\,j} \;=\; \sum_{i=1}^{n}\left[\left(\frac{y_i(s_j)}{\gamma(s_j)}\right)\left(\frac{y_i(\overline{S_{NK}})}{\gamma(\overline{S_{NK}})}\right)\right] \qquad \text{(E2)}$$

$$\gamma(s_j) \;=\; \sqrt{\sum_{i=1}^{n}(y_i(s_j))^2}$$

$y_i(s_j)$: Intensity of the jth spectrum (s_j) at wavenumber i

s_j: single spectrum from one sample (SARS-CoV-2 or Influenza) of a specific batch (Data Set 1 OR Data Set 2) from a specific replicate (day R1 – R4)

$$\gamma(\overline{S_{NK}}) \;=\; \sqrt{\sum_{i=1}^{n}(y_i\left(\overline{S_{NK}}\right))^2}$$

$y_i(\overline{S_{NK}})$: Intensity of the average negative control spectrum $\overline{S_{NK}}$ at wavenumber i

$\overline{S_{NK}}$: average negative control spectrum, we always use the negative control from the same batch and day, that the spectrum s_j was taken from

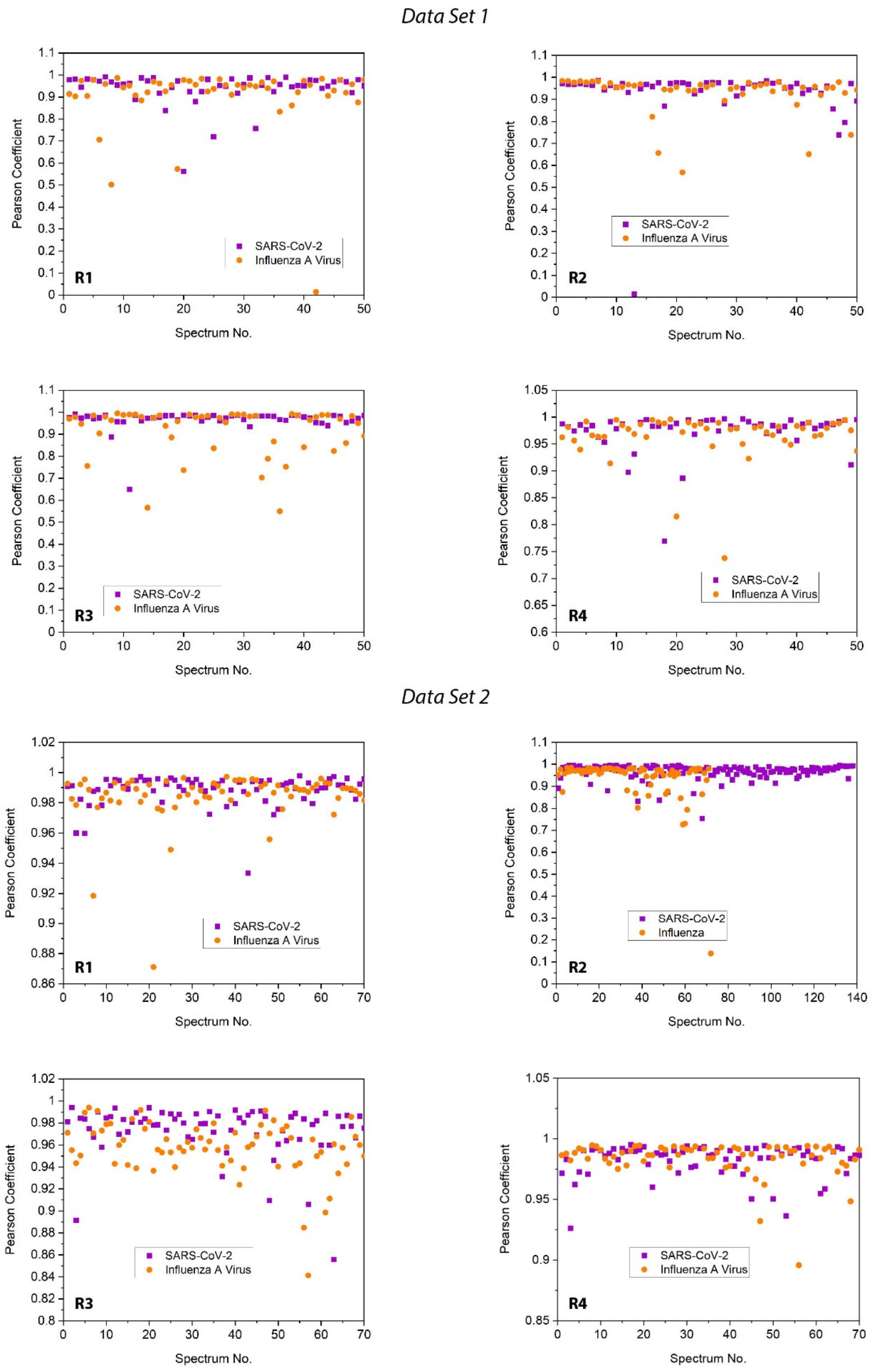

Figure S5. Distribution of the Pearson coefficients for all SARS-CoV-2 and Influenza A virus samples, for all replicates and for both data sets.

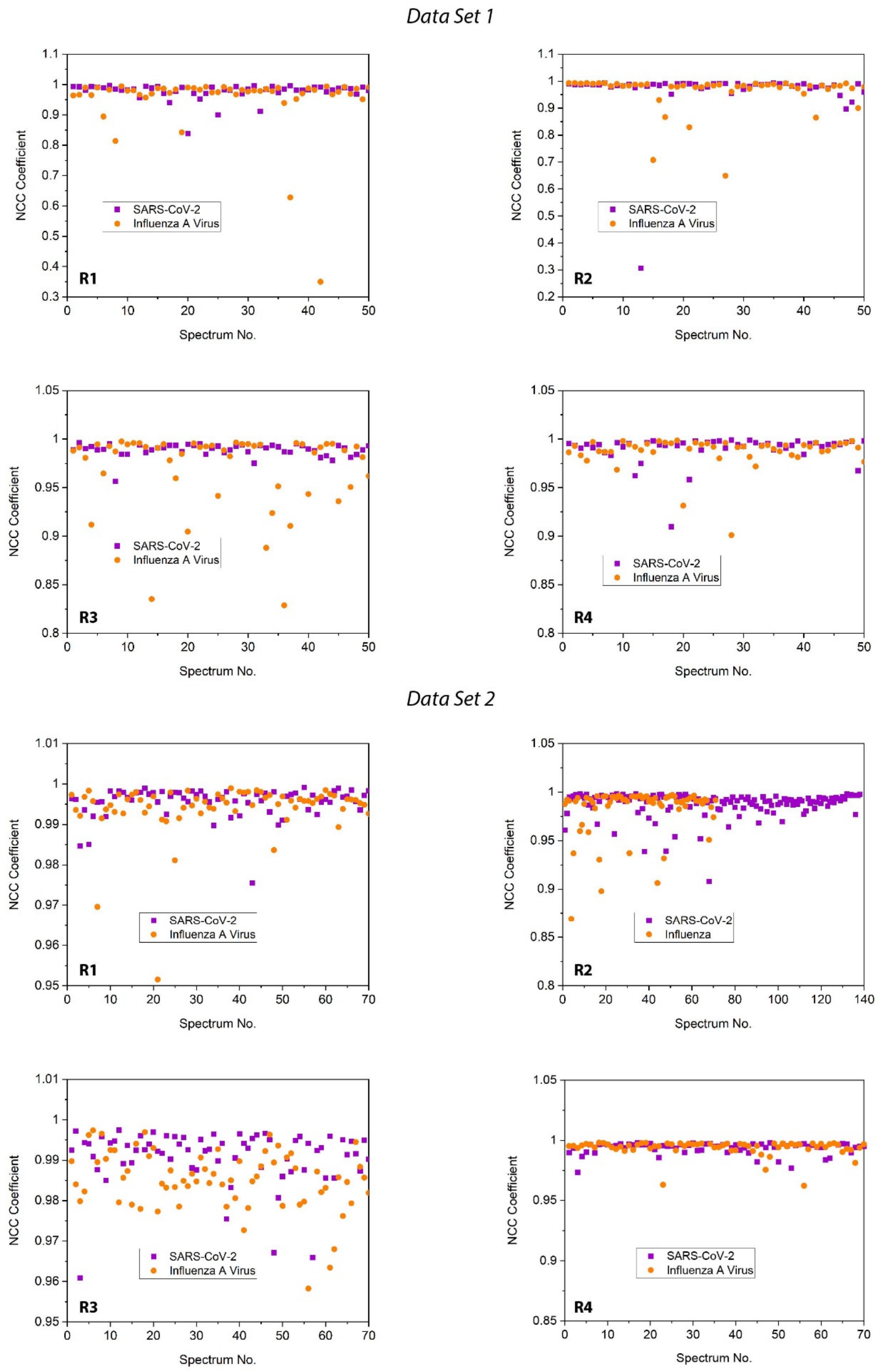

Figure S6. Distribution of the normalized cross correlation (NCC) coefficients for all SARS-CoV-2 and Influenza A virus samples, for all replicates and for both data sets.
